# Carbapenemases: epidemiology, detection and management in a changing global landscape

**DOI:** 10.1093/jacamr/dlag099

**Published:** 2026-06-08

**Authors:** Nenad Macesic, Patrick N A Harris, Yin Mo, Angela Gomez-Simmonds

**Affiliations:** Department of Infectious Diseases, The Alfred Hospital and School of Translational Medicine, Monash University, Level 1, Alfred Lane House, 55 Commercial Rd, Melbourne, VIC 3004, Australia; Centre to Impact AMR, Monash University, Melbourne, Australia; The University of Queensland, Faculty of Health, Medicine and Behavioural Sciences, UQ Centre for Clinical Research, Building 71/918, Herston, Australia; Herston Infectious Diseases Institute, Royal Brisbane and Women’s Hospital, Herston, Australia; Central Microbiology, Pathology Queensland, Royal Brisbane and Women’s Hospital, Herston, Australia; ADVANCE-ID, Saw Swee Hock School of Public Health, National University of Singapore, Singapore, Singapore; Mahidol-Oxford Tropical Medicine Research Unit, Faculty of Tropical Medicine, Mahidol University, Bangkok, Thailand; Oxford Centre for Global Health Research, Nuffield Department of Medicine, University of Oxford, Oxford, UK; Division of Infectious Disease, Department of Medicine, National University Hospital, Singapore, Singapore; Infectious Diseases Translational Research Programme, Yong Loo Lin School of Medicine, National University of Singapore, Singapore, Singapore; Division of Infectious Diseases, Department of Internal Medicine, UC Davis Health, Sacramento, CA, USA

## Abstract

Carbapenemase-producing Gram-negative bacteria are a growing threat to last-line beta-lactam (BL) therapy and are now established across Enterobacterales, *Pseudomonas aeruginosa* and *Acinetobacter baumannii*. Since the first reports in the 1980s, carbapenemase genes have disseminated internationally through plasmids, transposons and integrons and have become embedded in high-risk clones, with contemporary epidemiology dominated by *Klebsiella pneumoniae* carbapenemases (KPC), New Delhi metallo-beta-lactamase, Verona integron-encoded, imipenemase and oxacillinase (OXA)-type enzymes. The past decade has been marked by spread beyond hospitals, rising metallo-beta-lactamase (MBL) prevalence, and convergence of resistance with hypervirulence in some lineages. This Review summarizes carbapenemase classification, genetic contexts and epidemic clones. It describes regional distribution patterns alongside One Health drivers linking healthcare, community, animal and environmental reservoirs. We outline a pragmatic diagnostic framework spanning screening, confirmatory phenotypic assays, rapid lateral flow and molecular platforms, and whole genome sequencing for surveillance and outbreak investigation, emphasizing the clinical value of early mechanism identification for both infection control and targeted therapy. Treatment is reviewed in a mechanism-directed manner: newer BL/beta-lactamase inhibitor combinations are central for serine carbapenemases (including KPC and many OXA-48-like producers), whereas MBL producers require alternative strategies such as aztreonam-based combinations or cefiderocol. Options remain limited for carbapenemase-producing *P. aeruginosa* and *A. baumannii*, although sulbactam-durlobactam and pipeline agents are expanding the therapeutic landscape. We highlight the widening gap between disease burden and access to rapid diagnostics and novel therapies, particularly in high-burden low- and middle-income settings. Finally, we outline the bundled infection prevention and antimicrobial stewardship interventions needed to contain transmission and preserve the effectiveness of novel agents.

## Introduction

Carbapenemases are enzymes that inactivate most beta-lactam (BL) antibiotics, including carbapenems, and are now one of the most serious threats to last-line antimicrobial therapy. Since their first detection in the 1980s, carbapenemases have been identified across major Gram-negative pathogens such as Enterobacterales, *Pseudomonas aeruginosa* and *Acinetobacter baumannii*. Their frequent association with highly mobile plasmids has driven rapid international dissemination. This has prompted the World Health Organization to classify carbapenem-resistant Enterobacterales (CRE), *P. aeruginosa* and *A. baumannii* as critical priority pathogens.^1^ In 2019, there were >200 000 deaths globally attributed to carbapenem-resistant Gram-negative infections,^2^ with carbapenemase production as the dominant mechanism of resistance.

Carbapenems were introduced in the 1980s to treat infections caused by bacteria carrying extended-spectrum and AmpC beta-lactamases; however, carbapenemase activity had already been described in the 1970s.^3^ The first carbapenemase identified in Enterobacterales, the *Serratia marcescens* enzyme (SME), was reported in the 1980s.^4^ During the 1990s, carbapenemases emerged as a growing clinical concern, particularly among non-fermenting Gram-negative bacteria. This period saw the appearance of plasmid-mediated metallo-beta-lactamases (MBLs), including imipenemase-type (IMP) and Verona integron-encoded (VIM) enzymes in *Pseudomonas* spp., as well as oxacillinase (OXA)-type carbapenemases in *A. baumannii.*^5,6^ In the 2000s, two additional carbapenemase families became prominent in Enterobacterales. *Klebsiella pneumoniae* carbapenemases (KPC), first detected in the USA in 1996, spread rapidly and became endemic in multiple regions, including the USA, Israel, Italy, Greece and South America.^7,8^ New Delhi MBL (NDM) was first detected in 2008 in a returned Swedish patient and was subsequently shown to be widespread across South Asia.^9–11^

Since the 2010s, the global epidemiology of carbapenemases has been dominated by five major enzyme classes: KPC, NDM, VIM, IMP and OXA.^12^ Each has been associated with major outbreaks and the establishment of regional endemicity. By the 2020s, extensive international travel, plasmid-mediated gene flow and gaps in the consistent implementation of infection prevention and control measures have driven widespread global dissemination of carbapenemase-producing organisms (CPOs) (see Table 1 for terminology used in this Review), often extending well beyond their original geographic boundaries. The COVID-19 pandemic likely accelerated these trends through increased antimicrobial use and disruption of routine infection prevention practices.^13,14^ Of particular concern, MBLs are becoming increasingly prevalent, further restricting available treatment options. In parallel, carbapenemases have now been identified in hypervirulent lineages of *K. pneumoniae*, such as ST23, raising concern about the convergence of antimicrobial resistance (AMR) and virulence across multiple global settings.^15,16^

**Table 1.** Terminology of carbapenemase-producing and carbapenem-resistant Gram-negative organisms used in manuscript

Term	Acronym	Definitions
Carbapenemase-producing organism	CPO	Gram-negative organisms (Enterobacterales, *P. aeruginosa* and *A. baumannii*) producing an acquired carbapenemase
Carbapenemase-producing Enterobacterales	CPE	Enterobacterales producing an acquired carbapenemase
Carbapenem-resistant Enterobacterales	CRE	Enterobacterales that are phenotypically resistant to any carbapenem (including if underlying mechanism is not known)
Carbapenemase-producing *P. aeruginosa*	CPPA	*P. aeruginosa* producing an acquired carbapenemase
Carbapenem-resistant *P. aeruginosa*	CRPA	*P. aeruginosa* that is phenotypically resistant to any carbapenem (including if underlying mechanism is not known)
*P. aeruginosa* with difficult-to-treat resistance	DTR-PA	*P. aeruginosa* with treatment-limiting resistance to all traditional beta-lactams (including carbapenems) and fluoroquinolones
Carbapenem-resistant *A. baumannii*	CRAB	*A. baumannii* that is phenotypically resistant to any carbapenem (including if underlying mechanism is not known)

The expanding reach of CPOs has resulted in major clinical and economic consequences. These infections cause the same syndromes as those due to susceptible Gram-negative bacteria, including urinary tract infection, hospital-acquired and ventilator-associated pneumonia, intra-abdominal infection and bloodstream infection.^17–19^ However, outcomes are considerably worse and have varied between studies depending on clinical syndrome, carbapenemase type, patient population and setting.^20–22^ Across multiple studies, the 30-day mortality for CPO bloodstream infection ranges from 26% to 44%,^23,24^ almost double that of infections with carbapenem-susceptible organisms. The type of carbapenemase may affect outcomes but results have been mixed with some studies showing higher mortality rates with MBL-producers,^23,25^ while others have shown higher mortality for KPC-producers.^25^ CP-*P. aeruginosa* had a 30-day mortality of 22% for all infection types,^26^ while CP-*A. baumannii* bloodstream infection had a 28-day mortality rate of 53% in intensive care unit patients.^27^

In addition to the considerable morbidity and mortality, CPO infections also cause a substantial burden on health systems. A recent Japanese study noted 42.1% longer length of stay and 50.4% higher costs in patients with CPO infections.^28^ CPO colonization alone has been associated with six times higher mean costs during an outbreak of OXA-181-producing *Escherichia coli* in Australia.^29^ CPO outbreaks also carry significant costs: an English outbreak of NDM-producing *K. pneumoniae* implicating 40 patients cost 1.1M Euros over 10 months.^30,31^

Despite these challenges, recent years have seen major advances in our understanding of the pathogenesis and epidemiology of CPOs, alongside improvements in diagnostic methods, therapeutic strategies and infection prevention frameworks.^32^ This Review synthesizes these developments and provides a contemporary framework for understanding carbapenemases, their emergence and their clinical consequences. We focus specifically on acquired carbapenemases and the organisms in which they have the greatest impact: Enterobacterales, *P. aeruginosa* and *A. baumannii*.

## Classification of carbapenemases

**Figure 1.**
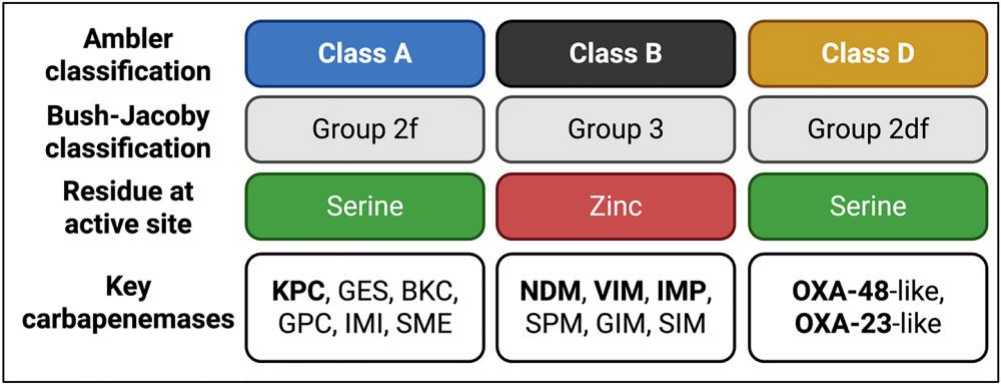
Overview of carbapenemase classification. Carbapenemases are classified by the Ambler molecular scheme and Bush–Jacoby functional classification into sierine beta-lactamases (Classes A and D) and MBLs (Class B), with representative clinically important families including KPC, MBLs (NDM, VIM, IMP), and oxacillinases (OXA-48-like and OXA-23-like) (shown in bold). Created with BioRender.com.

Beta-lactamases, including carbapenemases, are classified using two complementary frameworks: the Ambler molecular classification and the Bush–Jacoby functional grouping (Figure 1 and Table 2). The Ambler scheme organizes enzymes into Classes A, B, C and D based on amino acid sequence and overall protein structure.^33^ Carbapenemases arise from three of these classes. Class A and Class D enzymes are serine beta-lactamases that use an active-site serine to hydrolyse BLs, while Class B enzymes are MBLs that depend on one or two zinc ions at the catalytic site. These molecular categories reflect fundamental mechanistic differences: serine beta-lactamases hydrolyse BLs through formation and breakdown of an acyl-enzyme intermediate, whereas MBLs rely on zinc-mediated water activation to catalyse hydrolysis without forming a covalent intermediate.

**Table 2.** Characteristics of key carbapenemase families

Enzyme/ambler class	Origin and first reports	Key variants	Main clones/hosts	Plasmids/mobile elements	Notes
KPC (Class A)	USA, 1996; early 2000s expansion (incl. *K. pneumoniae* ST258/CG258)	>150 variants (KPC-2, KPC-3 most common); inhibitor-resistant variants (e.g. D179Y) emerging	*K. pneumoniae* ST258, ST307, ST147, ST11; spread to *E. coli*, *Enterobacter*; rare in *P. aeruginosa*, *A. baumannii*	Tn4401 on IncFIIk; also IncN, IncP	Major global driver of CRE; increasing resistance to beta-lactam/beta-lactamase inhibitor combinations
NDM (Class B MBL)	2008; first described in a returned Swedish patient (linked to South Asia)	>50 variants (NDM-1, NDM-5 common)	*E. coli* (ST131), *K. pneumoniae* ST11, ST147; also *P. aeruginosa*, *A. baumannii*	IncA/C, IncF, IncX3 plasmids; mobile elements	Community spread significant; dominant MBL family in Enterobacterales in many settings
VIM (Class B MBL)	Europe, 1990s (*P. aeruginosa*)	VIM-1, VIM-2	*P. aeruginosa* ST235, ST111; *K. pneumoniae* in Greece/Italy	Integrons on plasmids	VIM-2 dominant in *P. aeruginosa* globally; regional outbreaks persist
IMP (Class B MBL)	Japan, 1990s (*P. aeruginosa*)	IMP-1, IMP-4, IMP-7, IMP-8, IMP-26, IMP-27	*P. aeruginosa*, Enterobacterales	Integrons; plasmid-mediated	Regional endemicity with increasing global spread
OXA-48-like (Class D; Enterobacterales)	Türkiye, 2001 (*K. pneumoniae*)	OXA-48 group (e.g. OXA-48, OXA-244); OXA-181-like (incl. OXA-232); OXA-436 cluster	Predominantly Enterobacterales: *K. pneumoniae* (high-risk STs), *E. coli* ST131	Tn1999 (IS1999-flanked) on conserved IncL/M ‘pOXA-48’ plasmid; mosaic derivatives occur	Diverse phenotypes and lab detection challenges; successful plasmid maintenance/stability supports dissemination
Acquired OXAs in *A. baumannii* (Class D; CRAB-associated)	Emerged as major concern in *A. baumannii* in the 1980s–1990s	OXA-23-like, OXA-40-like (OXA-24/40), OXA-58-like; also OXA-143-like, OXA-235-like	*A. baumannii* international clones, especially IC2 (also IC5, IC7 reported)	Plasmid- or transposon-borne OXA genes; ISAba1 (and related IS) provides strong promoters and mobilization between plasmid/chromosome	Carbapenem resistance in *A. baumannii* commonly reflects acquired OXA + promoter effects (ISAba1) driving high-level resistance

The Bush–Jacoby functional system provides a complementary view by grouping beta-lactamases according to their substrate hydrolysis profiles and inhibitory susceptibility.^34,35^ Within this system, serine carbapenemases are encompassed in Group 2f for Class A enzymes and Group 2df for Class D enzymes. While Group 2f enzymes typically confer robust hydrolysis of carbapenems in addition to penicillins and cephalosporins, Class D OXA-type enzymes in Group 2df exhibit weaker but clinically significant carbapenem-hydrolysing activity that often requires additional permeability defects or co-resistance mechanisms to result in high-level carbapenem resistance. Class B MBLs are placed in Group 3, defined by their broad activity against carbapenems, limited hydrolysis of monobactams and inhibition by metal chelators such as ethylenediaminetetraacetic acid (EDTA).^34^

Although these carbapenemase classes differ markedly in structure, mechanism and functional properties, all produce clinically significant carbapenem resistance.^36^ This framework underpins the detailed discussion of individual carbapenemase families in the sections that follow.

## Major carbapenemase families

### Class A carbapenemases

Among Class A enzymes, KPC is the dominant acquired carbapenemase driving global spread. KPC enzymes have broad activity against penicillins, cephalosporins and carbapenems, supported by an active-site configuration that accommodates bulky substrates such as carbapenems. The global spread of KPC has been driven by its two predominant variants, KPC-2 and KPC-3, which differ by a single amino acid.^12^ These are found in a wide variety of Gram-negative bacteria including hospital-associated Enterobacterales, *Salmonella* spp., *P. aeruginosa*, and *A. baumannii.*^37,38^ KPC-4 is a less common variant that has been identified predominantly in *Enterobacter* spp. and *K. pneumoniae*.^39^ KPC-4 differs from KPC-2 by two amino acids and has decreased activity against carbapenems but an increased rate of ceftazidime hydrolysis.^40^

**Figure 2.**
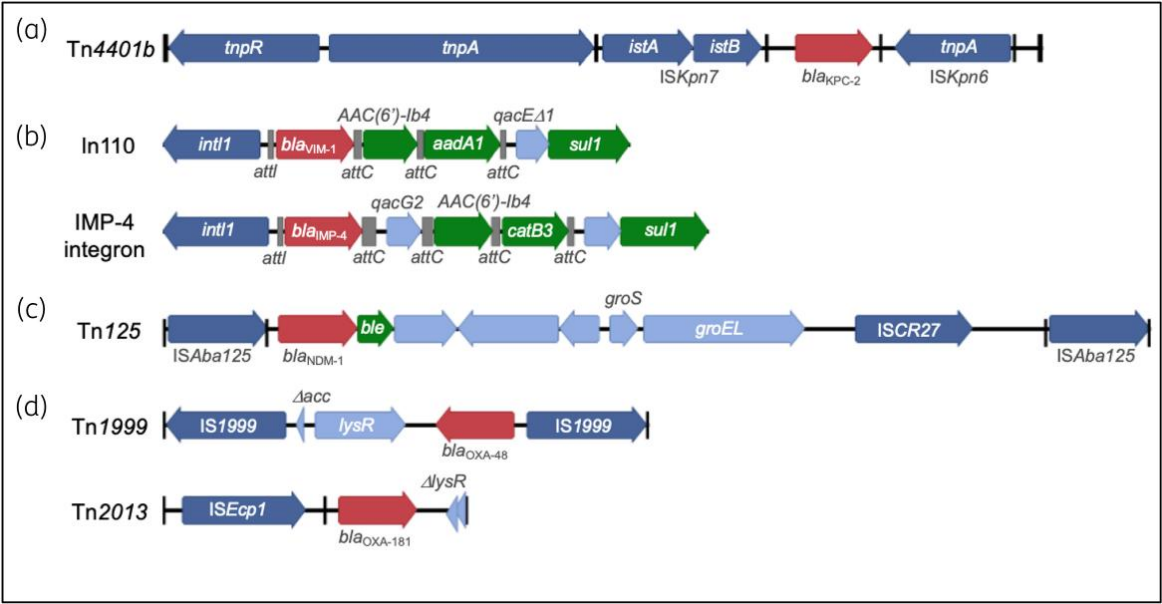
Genetic structures associated with transmission of globally widespread carbapenemase genes. (a) Variants of Tn4401 comprise the predominant genetic context for *bla*_KPC_, including Tn4401b shown here, where the deletion region (68–255 bp) upstream of *bla*_KPC_ found in other Tn4401 variants is marked by an open red rectangle. (b) Examples of Class I integrons associated with carriage of *bla*_VIM_ (In110) and *bla*_IMP_ (IMP-4 integron). (c) Tn125 transposon thought to be involved in initial mobilization of *bla*_NDM_ along with its embedded IS*CR27* element. (d) *bla*_OXA-48_ is typically associated with Tn1999 and found within the highly conserved plasmid pOXA-48, whereas *bla*_OXA-181_ is carried by Tn2013. Carbapenemase genes are shown in red, other antibiotic resistance genes in orange, and mobile genetic elements in dark blue; inverted repeats are indicated by black rectangles, and other structures are marked.

The success of KPC is closely linked to its genetic context. *bla*_KPC_ is most often embedded in the Tn3-type transposon Tn4401, which carries flanking insertion sequences and its own transposition machinery (Figure 2a). Variants of Tn4401 isoforms have been identified that predominantly differ by deletions in the region immediately upstream of the *bla*_KPC_ gene, which includes the promoters P1 and P2, leading to isoform-specific differences in *bla*_KPC_ expression and carbapenem resistance.^41,42^ Tn4401 inserts into a wide variety of conjugative plasmids.^43–45^ The plasmids most frequently encoding *bla*_KPC_ belong to the relatively narrow host range IncF family. This includes IncFIB(pQkil) and IncFIB/FII(K)-type plasmids, which are conserved within ST258 lineages and likely played an important role in the global spread of CRE.^46^ In contrast, broad host range plasmids, such as IncN, IncC and IncL/M plasmids, have been shown to contribute to the transmission of *bla*_KPC_ between different bacterial strains and species.^47^ Less commonly, *bla*_KPC_ is found in alternative structures called NTE_KPC_ (‘non-Tn4401 elements’) derived from portions of Tn4401 as well as other transposons and mobile genetic elements, facilitating adaptation to different hosts.^48^

Other acquired Class A carbapenemases, although less prevalent, follow a similar pattern of plasmid-mediated spread. Guiana extended-spectrum beta-lactamases (GES) are typically located in the variable regions of Class 1 (and occasionally Class 3) integrons on transferable plasmids. Most GES variants confer an ESBL phenotype. However, a subset including GES-2, GES-3, GES-5 and GES-16 has activity against carbapenems.^49^ Most of these variants have G170S or G170N substitutions resulting in an increased rate of hydrolysis for carbapenems.^50^ GES variants also confer problematic resistance to the BL/beta-lactamase inhibitor (BLI) combination antibiotics ceftolozane/tazobactam and imipenem/relebactam in MBL-negative *P. aeruginosa.*^51,52^ Recently described plasmid-encoded enzymes such as Brazilian *Klebsiella* carbapenemase-1 and Greek *Proteus mirabilis* carbapenemase-1, probably derived from environmental species, illustrate the ongoing recruitment of new Class A carbapenemases into clinically important plasmid backgrounds. In contrast, chromosomal Class A enzymes such as SME, imipenem-hydrolysing beta-lactamase (IMI)/NmcA and *Serratia fonticola* carbapenemase-1 remain largely confined to their native hosts and thus far have demonstrated limited transmission to other bacteria.^53^

### Class B carbapenemases (MBLs)

Class B carbapenemases comprise the zinc-dependent MBLs, with the most important examples being NDM, IMP and VIM in subclass B1. These enzymes hydrolyse almost all BLs, including carbapenems, but have limited activity against monobactams. Their spread has been enabled by association with integrons, transposons and broad-host-range plasmids that circulate widely among Enterobacterales, *P. aeruginosa* and *A. baumannii*.

IMP and VIM enzymes were the first transmissible MBLs to be identified. Both *bla*_IMP_ and *bla*_VIM_ are usually located within Class 1 integron gene cassettes that can be embedded either in the chromosome or in diverse plasmids such as IncC, IncL/M, IncHI2 and IncN-type plasmids (Figure 2b). This architecture allows rapid assembly of multidrug resistance regions where carbapenemase genes sit alongside resistance determinants for aminoglycosides, sulphonamides and other classes. Most IMP variants have been limited to specific regions, but IMP-1, IMP-4, IMP-7, IMP-8 and IMP-13 have achieved widespread geographical distribution.^54,55^ There are a large number of known VIM variants, which can be grouped into several sublineages based on their similarity to *bla*_VIM-1_, *bla*_VIM-2_, *bla*_VIM-5_ and *bla*_VIM-13_.^56,57^ While VIM enzymes are encountered mostly in Europe and Northern Africa in *K. pneumoniae*, *Enterobacter* spp., and other Enterobacterales, VIM-2 has been shown to be globally widespread in *P. aeruginosa*.^58,59^

NDM represents the most recently identified and now one of the most prevalent MBLs. NDM's typical genetic context includes the complete or truncated insertion sequence ISAba125 immediately upstream of *bla*_NDM_ and the adjacent bleomycin resistance gene *ble*_MBL_. ISAba125 has been shown to provide a -35 promoter involved in the expression of *bla*_NDM._^60^ An IS*CR* element, IS*CR27,* located downstream of *bla*_NDM_, may have contributed to its initial mobilization from *Acinetobacter* species. This was followed by fusion with ISAba125 and acquisition of a second copy of ISAba125 downstream of *bla*_NDM_ to form the composite transposon Tn125 associated with the spread of NDM among Enterobacterales and other Gram-negative bacteria (Figure 2c).^45,61^ More recently, acquisition by composite transposons and other mobile genetic elements has contributed to its ongoing dissemination. These elements have been mobilized onto a broad array of plasmid backbones rather than a single dominant replicon, contributing to the extraordinarily wide host range of NDM, from *K. pneumoniae* and *E. coli* to numerous other Enterobacterales and non-fermenters.^61^ To date, 59 NDM variants have been reported, which vary by fewer than 5 amino acids; however, differences in carbapenemase activity have been noted between them.^62,63^ Notably, while most beta-lactamases (including other MBLs) are typically secreted into the periplasmic space, NDM has a lipid moiety that enables anchoring to the outer membrane of Gram-negative bacteria as well as secretion in outer membrane vesicles.^64^ This adaptation is also thought to enhance NDM stability in the setting of zinc deprivation.

Several other plasmid-encoded B1 MBLs, such as German imipenemase, São Paulo MBL (SPM) and Seoul imipenemase, have emerged in specific regions.^65^ They are also carried by Class 1 integrons on transferable plasmids. Chromosomally encoded B2 and B3 enzymes (e.g. L1 MBL in *Stenotrophomonas maltophilia* and GOB-1 in *Elizabethkingia meningoseptica*) are important causes of intrinsic carbapenem resistance in the relevant species but have not mobilized into mobile genetic elements.^66^

### Class D carbapenemases (OXA enzymes)

Class D carbapenemases, or OXA-type enzymes, constitute the third major acquired carbapenemase family found in both *A. baumannii* and Enterobacterales. These enzymes were named OXAs because they hydrolysed oxacillin more readily than benzylpenicillin.^67,68^ However, extended-spectrum OXA variants were identified soon after in *P. aeruginosa*, followed by OXA carbapenemases in *A. baumannii* in the 1980s–1990s.^69^

All *A. baumannii* encode an intrinsic, chromosomally-encoded variant of OXA-51 with weak carbapenem hydrolysing activity that typically does not confer carbapenem resistance.^5,70^ However, OXA-51 overexpression due to insertion of an upstream ISAba9 and its associated promoter has been shown to increase carbapenem minimum inhibitory concentrations (MICs) into the resistant range.^71^ More commonly, carbapenem resistance in *A. baumannii* arises through acquisition of plasmid- or transposon-borne *bla*_OXA_ genes, particularly variants of *bla*_OXA-23_, *bla*_OXA-40_, *bla*_OXA-58_, *bla*_OXA-143_ and *bla*_OXA-235_. These genes are frequently associated with insertion sequences such as ISAba1, which can provide strong promoters when inserted upstream and markedly increase gene expression.^71,72^ As a result, even enzymes with relatively modest catalytic efficiency for carbapenems can confer high-level resistance when overexpressed. The same insertion sequences also promote movement between plasmids and chromosomes, supporting clonal expansion and horizontal transfer within *A. baumannii* populations and occasionally into other species (e.g. *P. mirabilis* and *E. coli*).

In Enterobacterales, OXA-48-like enzymes have become major contributors to carbapenem-resistant infections. OXA-48 appears to have originated from water-borne *Shewanella* species and is now found predominantly within variants of the composite transposon Tn1999, which was likely involved in the initial capture and mobilization of *bla*_OXA-48_ from this genus.^73,74^ Tn1999 consists of *bla*_OXA-48_ bracketed by two copies of IS1999, an arrangement that facilitates insertion into conjugative plasmids (Figure 2d). A single, highly conserved IncL/M plasmid, often referred to as pOXA-48 has been especially successful at disseminating *bla*_OXA-48_ globally and shows a strong association with high-risk *K. pneumoniae* sequence types.^75^ Mosaic derivatives of Tn1999 can incorporate additional resistance genes, while plasmid-encoded toxin–antitoxin systems and other stability mechanisms promote maintenance of pOXA-48 in clinical strains. Though closely related, evidence suggests that OXA-181 was acquired independently from *Shewanella xiamenensis*, likely through association with IS*Ecp1*, which was then embedded within Tn2013 (Figure 2d).^76^

Three clusters of OXA-48-like enzymes have been identified; variants with high amino acid homology to OXA-48 (including OXA-162, OXA-244, OXA-245 and OXA-519), OXA-181-like enzymes (including OXA-232 and OXA-484), and OXA-436, which clusters separately from the other enzymes.^72,77^ OXA-48-like enzymes share a compact active site that favours deacylation of carbapenems and display variable activity against expanded-spectrum cephalosporins, leading to diverse phenotypic profiles that can complicate laboratory detection. Together, acquired OXA carbapenemases illustrate how moderate intrinsic catalytic activity can be amplified by strong promoters, stable plasmids and successful bacterial hosts to produce major clinical AMR problems.

## Epidemic carbapenemase-producing clones

The global dissemination of carbapenemases has been driven not only by mobile genetic elements but also by a limited number of highly successful bacterial clones that have repeatedly acquired, stabilized and propagated carbapenemase genes. This phenomenon is most clearly illustrated by *K. pneumoniae*, in which several epidemic lineages have played a central role in the worldwide spread of KPC, NDM and OXA-type carbapenemases.

The spread of KPC carbapenemases has been dominated by *K. pneumoniae* clonal group 258 (CG258), which includes sequence types ST258, ST11, ST340 and ST512. ST258, the archetypal KPC-producing clone, is most associated with KPC-2 and KPC-3 encoded on IncF plasmids, particularly those with pKpQIL or pKPN backbones.^48^ Early genomic investigations linked ST258 to hospital and regional outbreaks in Israel, Italy, Greece and the northeastern United States in the early 2000s. Subsequent comparative genomics demonstrated that ST258 consists of at least two major clades that arose through large-scale recombination events involving the capsule synthesis locus.^78^ These recombination events generated antigenic diversity and were followed by ongoing adaptive variation at the capsule locus, likely contributing to immune evasion, persistence and transmission.^79,80^ Other CG258 clones have also been important. ST340 has disseminated widely across Europe, South America, Southeast Asia and Africa, driven in part by expansion of the KL15 sublineage, while ST512 has been reported predominantly in Europe.^81^

ST11 represents a closely related but distinct CG258 lineage that has achieved prominence in East Asia. It has remained the dominant KPC-producing clone in China since the early 2000s and has increasingly been associated with additional carbapenemases, including NDM and OXA-48-like enzymes.^82^ Compared with ST258, ST11 displays greater genetic diversity and is more frequently enriched for virulence-associated loci, including the yersiniabactin siderophore system.^83^ Of particular concern, ST11 strains carrying classical hypervirulence plasmids such as pLVPK or pK2044 have been reported, demonstrating convergence of multidrug resistance and hypervirulence within a globally disseminated clone.^84^

Beyond CG258, several *K. pneumoniae* lineages have emerged as important epidemic carbapenemase producers. ST147 arose in Europe as an ESBL-producing clone and subsequently acquired a wide range of carbapenemases, including VIM, KPC, NDM and OXA-48-like enzymes.^85^ It is now globally distributed and represents a dominant carbapenem-resistant lineage in Africa, predominantly North Africa.^86^ ST307 was initially described as a CTX-M-15-producing clone and has since expanded internationally, frequently carrying KPC or OXA-48-like carbapenemases in combination with ESBLs.^87,88^ These lineages highlight the ability of successful ESBL-producing clones to transition into carbapenemase producers following acquisition of carbapenemase-carrying mobile genetic elements.

A similar pattern has been observed in *E. coli*. The globally dominant ST131 clone, best known for dissemination of CTX-M-15 within the H30-Rx sublineage, was among the first *E. coli* lineages shown to acquire NDM following its emergence in South Asia.^89^ Since then, ST131 producing OXA-48-like enzymes and multiple NDM variants has been reported worldwide. Other *E. coli* lineages with epidemic potential include ST167 and ST410. ST167 contributed early to the spread of NDM in China, often in association with IncX3 plasmids,^90^ and has since disseminated across multiple continents through expansion of its C2 subclade. ST410 emerged as a widely distributed ESBL-producing lineage following a *fimH* allele shift and has subsequently acquired KPC, NDM and OXA-48-like enzymes, frequently in combination with CTX-M-15.^91,92^ Recent reports of ST410 strains co-harbouring OXA-181 and NDM-5 underscore its capacity to accumulate multiple carbapenemase genes.^86^ Additional *E. coli* sequence types, including ST10, ST38 and ST405, have also been associated with carbapenemase carriage, although with more limited global impact.

Within the *Enterobacter cloacae* complex, several clones have contributed to regional and international dissemination of carbapenemase genes, although resistance remains more polyclonal than in *K. pneumoniae*. *Enterobacter hormaechei* ST78 was initially recognized as a widespread ESBL-producing clone and has since been linked to dissemination of KPC in North America and IMP, VIM, NDM and OXA-48-like enzymes across Asia, Europe and the Middle East.^93–95^ ST78 has been shown to acquire carbapenemase genes on diverse plasmid backbones, suggesting a high capacity for resistance plasmid uptake and maintenance. ST171 has been an important driver of KPC-3-producing *E. cloacae* complex in North America and has more recently been associated with OXA-48 and NDM-5.^96^ Other globally distributed *E. cloacae* complex clones associated with carbapenemase carriage include ST90, ST93, ST133, ST418 and *Enterobacter xiangfangensis* ST114.^97,98^

Fewer epidemic carbapenemase-producing clones have been described among non-fermenting Gram-negative bacteria, but several remain clinically important. In *P. aeruginosa*, ST235 is a globally distributed multidrug-resistant lineage that emerged in the 1980s and has demonstrated a remarkable ability to acquire diverse carbapenemases, including KPC, GES, VIM, IMP and NDM.^99^ ST235 has been implicated in multiple outbreaks, particularly involving VIM-2 and NDM-1 in Europe and Asia. Other internationally distributed *P. aeruginosa* clones associated with carbapenemase production include ST111, ST175, ST233 and ST308.^100^

In *A. baumannii*, epidemic lineages are commonly defined by international clone (IC) designations.^101^ IC2 has been the dominant multidrug-resistant lineage worldwide and is strongly associated with acquired Class D carbapenemases, particularly OXA-23, OXA-24/40 and OXA-58. This clone contributed substantially to the rise in carbapenem-resistant *A. baumannii* (CRAB) during the 2000s. Other lineages, including IC5 and IC7, have also been linked to OXA-23 dissemination and have achieved wide geographic spread in Europe and South America.

## Co-production of multiple carbapenemases

Increasingly, carbapenemase transmission in Enterobacterales is not limited to single enzymes within dominant clones. Reports of dual- or multi-carbapenemase producers have risen over the past decade across diverse species, including *Klebsiella*, *Enterobacter* and *Citrobacter* spp., reflecting horizontal gene transfer rather than clonal spread alone.^102–104^ The *Citrobacter freundii* species complex has emerged as an important reservoir in the healthcare environment, as well as clinical isolates.^105,106^ It frequently carries OXA-48-like, KPC, NDM and VIM enzymes and can acquire combinations such as KPC-2 plus NDM-1 on transferable plasmids.^104,106–108^ Multispecies dissemination of isolates co-harbouring *bla*_KPC_ and *bla*_NDM_, as well as other serine carbapenemase/MBL combinations has been documented in healthcare settings.^102,103^ These organisms often exhibit extensive multidrug resistance and reduced susceptibility to novel BL/BLI combinations, leaving few reliable therapeutic options and necessitating mechanism-directed regimens such as aztreonam-based combinations or cefiderocol. The emergence of multi-carbapenemase producers complicates both phenotypic detection, where multiple enzymes may mask each other's presence, and treatment selection.

## Global distribution of CPOs

**Figure 3.**
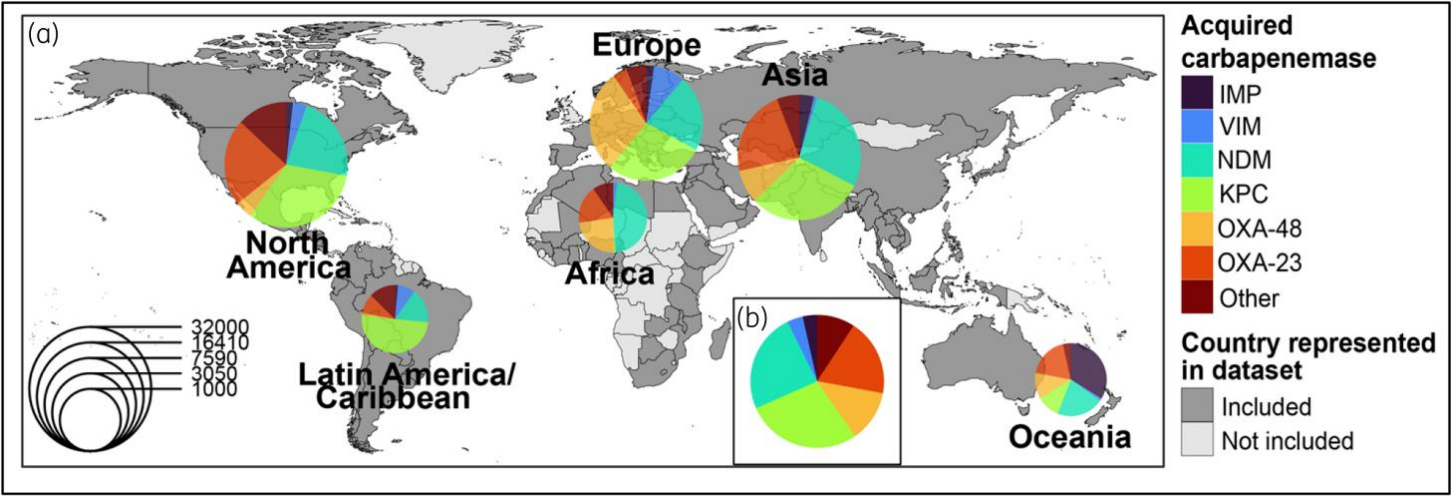
(a) Geographic and (b) overall distribution of genomes containing key acquired carbapenemases. Genomes containing acquired carbapenemases (*n* = 104 032) were downloaded from AllTheBacteria (date accessed: 2 October 2025). Metadata for corresponding BioSample identifiers were retrieved from the National Center for Biotechnology Information. These distributions reflect the composition of publicly-available sequenced genomes and are influenced by regional sequencing intensity, surveillance priorities and study design. They should therefore be interpreted as patterns of sequencing representation rather than estimates of population-level prevalence.

The global burden of CPOs reflects the combined effects of mobile resistance genes and their successful integration into high-risk bacterial lineages. While the preceding section focused on epidemic clones as key vehicles for dissemination, this section summarizes the resulting geographic patterns, which vary substantially between regions according to historical introductions, healthcare practices and regional connectivity (Figure 3).

### Asia and Oceania

The Asia-Pacific region reports some of the highest global CPO rates and harbours all major carbapenemase families, with substantial heterogeneity between subregions. In South Asia, including India and Pakistan, carbapenem resistance rates of 20%–30% have been reported in Enterobacterales and *P. aeruginosa*, while rates in *A. baumannii* frequently exceed 80%.^109^ NDM carbapenemases have historically predominated, with prevalence ranging from 15%–50% in some settings, although OXA-48-like carbapenemases have emerged as an increasingly important contributor and are often detected concurrently with NDM.^110–112^ Concerningly, high CPO rates have been noted in both hospital and community settings, including in the aquatic environment.^10^ South Asia is also linked to the Pacific through travel and medical tourism, with NDM-producing organisms reported in countries such as Fiji following importation.^113,114^

In East Asia, KPC carbapenemases are major contributors, particularly in China where they are strongly associated with *K. pneumoniae* ST11. MBLs also play an important role, with NDM increasingly reported in China and IMP carbapenemases having become endemic in Japan since their first description in the late 1990s.^54^ Southeast Asia demonstrates convergence of KPC and NDM carbapenemases, with relative predominance varying by country. For example, NDM predominates in Thailand, KPC in Indonesia and both are prominent in Singapore.^115^ Carbapenemase-producing *P. aeruginosa* and *A. baumannii* are highly prevalent in hospitals across the region. In *P. aeruginosa*, MBLs such as VIM and IMP are common and often associated with high-risk ICs including ST235.^99^ In *A. baumannii*, carbapenem resistance exceeds 80% in some countries and is largely driven by OXA-23-like carbapenemases.^116^

Oceania has comparatively low rates of carbapenem resistance. In Australia, IMP producers are endemic in some centres on the east coast, prior outbreaks of KPC producers have been documented and NDM carbapenemases now contribute an increasing proportion of cases.^117–119^ In New Zealand, most CPOs are linked to international travel.^120^ Data from Pacific Island nations remain limited, although available reports suggest endemic OXA-23- and NDM-producing *A. baumannii* in Fiji, with NDM also detected in Enterobacterales and *P. aeruginosa.*^113,121^

### Europe

The epidemiology of CPOs in Europe is highly heterogeneous and follows a pronounced gradient from north to south and west to east.^15^ In several Southern and Eastern European countries, more than 50% of *K. pneumoniae* bloodstream isolates are carbapenem resistant, whereas rates remain below 1% in much of Northern Europe.^15^ Similar patterns are observed for *P. aeruginosa* and *A. baumannii*, with particularly high resistance rates in countries such as Italy, Greece, Russia and Türkiye.

Carbapenemase distribution varies by subregion and host species. In Enterobacterales, KPC has historically been the dominant carbapenemase in Southern Europe, although NDM is playing an increasing role, including in major outbreaks and rising prevalence in countries such as Greece.^122–124^ OXA-48-like carbapenemases, first described in Türkiye, are now dominant across the Balkans and account for the majority of carbapenemase-producing Enterobacterales (CPE) in several countries.^123^ VIM carbapenemases were first described in Italy and continue to be an important cause of resistance in *P. aeruginosa.*^6,125^

### Africa and the Middle East

Africa and the Middle East experience high CPO burden, with substantial regional variation. In the Middle East, OXA-48 and NDM carbapenemases are widespread and continue to increase in prevalence.^126^ This reflects both regional dissemination of OXA-48 from Türkiye and repeated importation of NDM from South Asia. Healthcare-associated outbreaks have played an important role in spread, and several countries in the region have experienced sustained endemicity.^38,127^ In addition, the region has also experienced significant conflict-related healthcare disruption (e.g. Syria, Yemen, Iraq), resulting in displacement of millions of people and facilitating CPO spread across borders, both to neighbouring countries and into low prevalence regions.^128,129^ Israel has a specific epidemiology, with KPC-producing *K. pneumoniae* ST258 causing an early epidemic and becoming established following importation from the USA.^130^ The region is a global hotspot for CRAB, which gained worldwide attention due to combat-related infections of OXA-23-producing *A. baumannii* noted in US veterans returning from Iraq.^131^

North Africa shares many epidemiological features with the Middle East.^77^ OXA-48 is considered endemic across much of the region and accounts for the majority of carbapenem-resistant Enterobacterales in countries such as Algeria, Libya and Tunisia.^132^ In contrast, NDM carbapenemases play a more prominent role in Egypt and Morocco. Prevalence of carbapenem resistance in *K. pneumoniae* reached 50.2% in Egypt and 65% in Sudan.^133^ High-risk clones such as *K. pneumoniae* ST101 are associated with OXA-48 dissemination, while globally distributed clones such as ST147 contribute to NDM spread.^133,134^

Data from sub-Saharan Africa remain limited outside South Africa, but available evidence indicates rapid increases in carbapenem resistance in hospital settings due to increasing carbapenem use since the 2010s and carbapenemase importation due to travel.^135^ NDM and OXA-48 predominate overall, with regional variation. In South Africa, OXA-48 is most frequently reported,^133,136–138^ whereas NDM carbapenemases appear more common in West Africa.^139^

### North America

In North America, KPC carbapenemases emerged rapidly in the USA during the 2000s and became endemic in several regions, largely driven by dissemination of *K. pneumoniae* ST258.^140,141^ Prevalence remains geographically heterogeneous, with higher rates in eastern states such as New York and Pennsylvania.^142^ CPO prevalence increased further during the COVID-19 pandemic. Although some reductions have occurred, rates remain above pre-pandemic levels.^143^ Recent surveillance suggests a shift in epidemiology, with recent reports showing NDM Enterobacterales starting to surpass KPC producers both in New York and countrywide.^144,145^ Other MBLs such as VIM and IMP are detected sporadically and in outbreak settings.^146,147^ CRAB remains a significant problem in the USA, accounting for one-third of *A. baumannii* infections. Canada reports lower overall prevalence of CPE, with KPC and NDM found in 46% and 29% of carbapenemase-producing isolates, respectively, in a recent study.^148^

### Latin America and the Caribbean

Latin America experienced substantial increases in carbapenem resistance during the 2010s, with reported rates of 10 to nearly 70% in *K. pneumoniae*.^149^ This increase was largely driven by dissemination of KPC carbapenemases within epidemic clones such as *K. pneumoniae* ST258 and ST11, leading to endemicity in countries including Brazil, Colombia and Argentina.^150^ During the COVID-19 pandemic, further increases were reported, alongside emergence of Enterobacterales carrying both KPC and MBLs,^149^ reflecting broader regional shifts towards NDM.^151^ Enterobacterales account for most carbapenemase producers in Latin America, but CRAB remains highly prevalent and is predominantly associated with OXA-23. Carbapenem-resistant *P. aeruginosa* isolates most often produce MBLs, particularly VIM and IMP, with SPM rarely reported in Brazil.^150,151^ Data from the Caribbean are limited. Regional surveys have identified KPC as the dominant carbapenemase, although in Cuba NDM-producing *K. pneumoniae* has been reported as the most frequent CPE.^152,153^

## Drivers of carbapenemase spread: a One Health perspective

**Figure 4.**
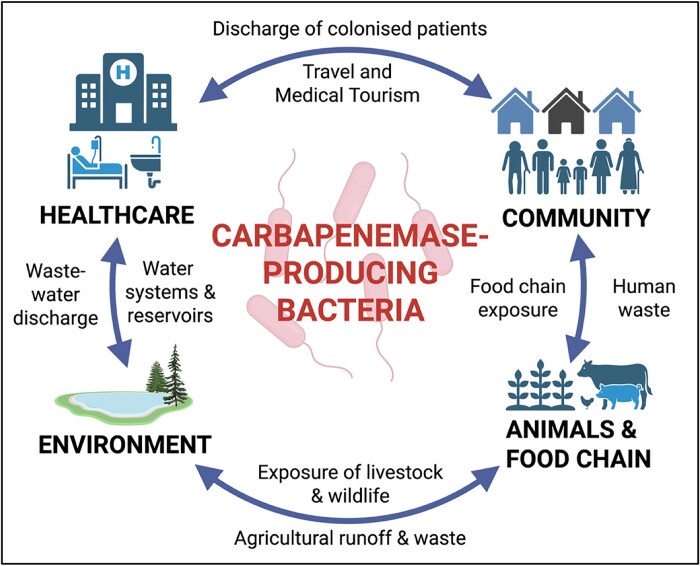
One Health drivers of carbapenemase spread. Carbapenemase dissemination is sustained by connections between healthcare, community, animal/agricultural and environmental domains. Created with BioRender.com.

The geographic patterns described above reflect more than clonal expansion and regional healthcare practices. Carbapenemase dissemination is sustained by interconnected reservoirs across human, animal and environmental domains.^154^ A One Health framework is therefore essential for understanding how carbapenemase genes persist, circulate and re-enter clinical settings, often beyond the reach of traditional infection prevention measures (Figure 4).

### Human reservoirs

Healthcare settings remain central to the dissemination of CPOs. Genomic epidemiology studies have demonstrated frequent within-hospital transmission and substantial interhospital spread. In the EuSCAPE study, more than half of participating hospitals showed evidence of local transmission, with movement of patients between institutions contributing to within-country spread.^155^ Hospitals also act as long-term reservoirs. Increasing attention has focused on the built environment, particularly sinks, drains and plumbing systems, where CPOs can persist in biofilms and cause transmission events to patients (e.g. through splashing).^156^ MBL-producers appear particularly well adapted to these aqueous niches.^157,158^ These observations have prompted renewed interest in sink decontamination strategies and redesign of clinical spaces, including adoption of ‘waterless’ intensive care unit models.^159,160^

Following acquisition in healthcare settings, colonized individuals may introduce CPOs into the community and vice versa. Community reservoirs are now well established in several regions.^161^ Studies from South Asia have reported community carriage rates of 15% in India and 9% in Bangladesh, while carriage rates of ∼6% have been described in Chile.^162^ A systematic review of community-associated or community-onset CRE infections reported proportions ranging from 7.7% to 29.5% globally and from 5.6% to 10.8% in the USA, underscoring the growing importance of non-hospital reservoirs.^163^ International travel and medical tourism further connect healthcare and community reservoirs across regions.^164^ The earliest reports of NDM involved travellers returning from South Asia, often following healthcare exposure.^9,11^ Recognition of this pathway has led to implementation of targeted screening policies for patients hospitalized abroad in many national and international guidelines.^165–167^ Travel-related importation has played a pivotal role in multiple major epidemiological shifts, including introduction of KPC-producing organisms into Israel from the USA, dissemination of OXA-48 from Türkiye into the Middle East and North Africa, and repeated introductions of NDM into Europe, Africa and Latin America.^168,169^

Not all colonized individuals progress to infection, and the risk of this transition is influenced by both host and pathogen factors. In a prospective genomic surveillance study of liver transplant recipients, colonization with CPE was significantly associated with subsequent invasive infection, whereas no infections occurred among patients colonized with non-carbapenemase-producing CRE.^170^ A large nationwide Israeli cohort study of nearly 7000 CPE carriers found that the overall risk of bloodstream infection was lower than previously reported in studies focused on high-risk populations, with a cumulative incidence of 2.4%.^171^ The risk of progression differed significantly by bacterial species (*E. coli* less than *K. pneumoniae*), and by clinical setting (higher rates among patients in intensive care and oncology/haematology wards). Of interest, the risk did not differ by carbapenemase type. Together, these findings suggest that the likelihood of progression from CPE colonization to infection is shaped by organism-level factors, host vulnerability and healthcare context rather than by the carbapenemase mechanism alone, with implications for risk stratification and the targeting of infection prevention interventions.

### Environmental reservoirs

Beyond healthcare settings, environmental compartments play a critical role in maintaining and amplifying carbapenemase dissemination and diversity. Hospital effluent discharged into municipal wastewater systems frequently contains CPOs and resistance genes, which can be detected downstream of healthcare facilities.^172,173^ Hospital water system microbiomes are also thought to promote gene transfer, accelerating transmission of carbapenemase genes.^174^ This has raised concerns about environmental amplification and has prompted investigation of on-site wastewater treatment to reduce release of resistant bacteria and antibiotic residues.^175,176^ Industrial sources also contribute. Pharmaceutical manufacturing effluents, particularly in parts of India and China with concentrated antibiotic production, have been associated with exceptionally high levels of antimicrobial residues and resistance genes, creating strong selective pressure for CPOs.^177,178^ More broadly, contamination of surface water and drinking water with human or animal waste represents a major transmission pathway. In South Asia, contaminated drinking water has been implicated in widespread NDM dissemination.^10^ A landmark 2011 survey detected NDM-producing bacteria in 4% of sampled drinking water sources and in nearly one third of seepage water samples.^10^ These pathways create a reinforcing cycle in which resistance genes of human origin enter water and soil, persist and diversify, and subsequently re-enter human populations through direct contact, food or drinking water.

### Animal reservoirs

Although carbapenems are not widely used in veterinary medicine, CPOs have been detected in a range of animal hosts, including livestock, aquaculture species and companion animals.^179,180^ Most reports involve food-producing animals such as poultry and swine, with detection of all major carbapenemase classes.^180^ Poultry production has been particularly implicated, including reports of NDM-producing Enterobacterales in Chinese poultry farms, reflecting patterns seen with other forms of AMR such as extended-spectrum beta-lactamases.^181^ Transmission through the food chain following contamination during production or processing is a key concern. Environmental exposure also links animal and human reservoirs. CPOs have been identified in scavenging and migratory birds that have contact with contaminated water or human waste,^182–184^ raising the possibility of long-distance dissemination. In one Australian study, IMP-4 carriage was detected in 40% of sampled gulls, highlighting the potential for wildlife to act as sentinels and vectors of environmental carbapenemase spread.^182^

## Diagnostics: phenotypic and genotypic approaches

**Figure 5.**
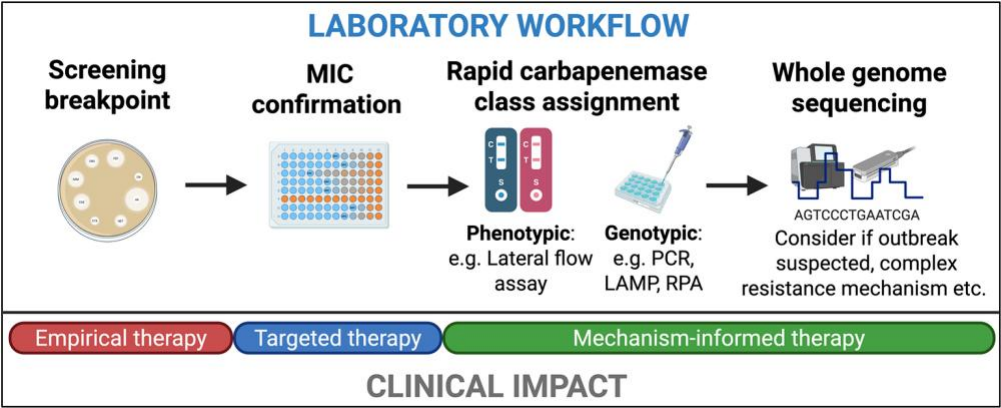
Laboratory detection of carbapenemases and clinical impact. Overview of laboratory approaches used to detect carbapenemase-producing Gram-negative bacteria and their downstream clinical implications. Reduced carbapenem susceptibility prompts phenotypic and/or rapid genotypic testing to identify carbapenemase activity and class. Results inform infection prevention measures, antimicrobial stewardship and selection of mechanism-directed therapy, with whole genome sequencing supporting surveillance and outbreak investigation. LAMP, loop-mediated isothermal amplification; MIC, minimum inhibitory concentration; PCR, polymerase chain reaction; RPA, recombinase polymerase amplification. Created with BioRender.com.

The One Health reservoirs described above sustain carbapenemase dissemination across healthcare, community, animal and environmental domains. Translating this understanding into control requires timely and accurate detection. There may be several reasons that justify the need to detect or characterize carbapenemases, including: (i) infection control purposes, i.e. detection of patient transmission events and outbreaks of strains with similar resistance mechanisms, (ii) clinical treatment, as clinical breakpoints may fail to detect the presence of some carbapenemases (especially OXA-48-like), potentially compromising antibiotic therapy, and (iii) surveillance at an institutional, national or international level to inform guidelines, drug development or policy. This need has become more acute as newer therapeutic options, particularly novel BL/BLI combinations, show activity that depends on the underlying carbapenemase class.^19^ Diagnostic approaches can be broadly divided into phenotypic methods, which infer carbapenemase activity from growth or hydrolysis, and genotypic methods, which detect carbapenemase genes directly (Figure 5). Most testing remains laboratory based, although point-of-care devices are increasingly being developed for direct-from-sample-detection at the (near) bedside.^185^

### Phenotypic detection methods

Phenotypic testing is typically initiated after routine susceptibility testing suggests reduced carbapenem susceptibility. Prior to 2010, Clinical and Laboratory Standards Institute (CLSI) clinical breakpoints for carbapenems were higher than current values and, as a consequence, did not reliably flag the presence of carbapenemase-producing isolates.^186^ Subsequent revisions, driven primarily by updated pharmacokinetic/pharmacodynamic exposure targets and wild-type MIC distribution data, lowered breakpoints substantially and improved their alignment with European Committee on Antimicrobial Susceptibility Testing (EUCAST) guidelines^187^ (Table 3). Although detection of resistance mechanisms is not the principal purpose of clinical breakpoints, the revised values now serve as more effective triggers for initiating carbapenemase-specific confirmatory testing.

**Table 3.** CLSI and EUCAST clinical breakpoints for carbapenems and Enterobacterales

Carbapenem	CLSI (S/I/R) mg/L	EUCAST (S/I^a^/R) mg/L	ECOFF (mg/L)
Imipenem	≤1/2/≥4	≤2/4/≥8^c^	0.5
Ertapenem	≤0.5/1/≥2	≤0.5/—/>0.5	0.064
Meropenem	≤1/2/≥4	≤2/2–4/≥8^b^	0.125

^a^EUCAST ‘I’ category = ‘Susceptible increased exposure’.

^b^Breakpoints applicable if meningitis is absent (for meningitis: ≤2 = S, >2 = R).

^c^Excludes *Morganellaceae;* ECOFF = epidemiological cut-off. Based upon EUCAST v15 document and CLSI M100 35th edition.

Current EUCAST guidelines recommend using a screening breakpoint for meropenem of MIC >0.125 mg/L (or <28 mm zone diameter if accompanied by resistance to piperacillin-tazobactam and/or temocillin, or <25 mm in all other cases), which corresponds to the epidemiological cut-off (ECOFF).^188^ Enterobacterales isolates with MICs below this value are unlikely to produce clinically significant carbapenemase. Given that members of the *Morganellaceae* family (e.g. *Proteus*, *Providencia*) have naturally occurring higher MIC distributions for imipenem, and with ertapenem showing poor specificity (e.g. through resistance resulting from ESBL/AmpC activity with porin changes without carbapenemase), meropenem is often used to provide the best trade-off between sensitivity and specificity for CPE detection.^189^

Detection of carbapenemases in *P. aeruginosa* is challenging because carbapenem resistance often arises through non-enzymatic mechanisms such as OprD loss and efflux upregulation,^190^ limiting the specificity of susceptibility-based screening. Nevertheless, acquired carbapenemases, most commonly VIM and NDM and less frequently GES, are increasingly reported. Because GES enzymes are often not included in commercial molecular or lateral flow assays, laboratories frequently rely on locally adapted screening algorithms.^191^ Screening algorithms incorporating resistance to meropenem or imipenem together with ceftazidime and cefepime perform well overall, although GES producers may be missed. In settings where GES is prevalent, inclusion of ceftolozane–tazobactam resistance can improve detection. In *A. baumannii*, carbapenem resistance is usually mediated by carbapenemases, most commonly OXA-type enzymes such as OXA-23 or MBLs including NDM. These isolates typically show high-level carbapenem resistance and screening breakpoints have not been developed.^188^

Once resistance to carbapenems has been detected by application of the screening breakpoints, confirmatory phenotypic tests can be used to demonstrate carbapenemase activity and, in some cases, provide presumptive class assignment. The CLSI initially recommended the modified Hodge test but it is no longer recommended because of poor performance.^192^

The carbapenem inactivation method (CIM) is a simple and low-cost phenotypic assay that detects carbapenemase activity through inactivation of a carbapenem disk. It shows good concordance with molecular methods for common carbapenemases, including KPC, NDM, OXA-48, VIM, IMP and OXA-23, in Enterobacterales and selected non-fermenters such as *P. aeruginosa.*^193^ Reduced sensitivity of the original CIM for some NDM- and OXA-48-like producers led to development of the modified CIM (mCIM), which has demonstrated high performance in multicentre studies, with reported sensitivity and specificity of ∼97%–99%.^194,195^ CIM and mCIM are inexpensive (<1 US dollar per test) and technically straightforward, making them suitable for routine use and resource-limited settings. However, false-positive results can occur with heavy inocula of isolates expressing weakly carbapenem-hydrolysing beta-lactamases, and performance is poor in *A. baumannii*, where alternative diagnostic approaches are required.^196^

Hydrolysis-based assays such as CarbaNP and its commercial derivatives provide rapid detection of carbapenemase activity through pH change following imipenem hydrolysis, with high specificity but poor performance for OXA-48-like or GES-producers.^197^ Inhibitor-based synergy tests using aminophenylboronic acid and EDTA can further support phenotypic differentiation of Class A (e.g. KPC) and Class B (e.g. NDM) carbapenemases, respectively. These tests do not detect OXA-type enzymes and are therefore most useful when combined with other indicators such as temocillin susceptibility or structured phenotypic algorithms.^198^ In an evaluation of 11 phenotypic methods across multiple laboratories in the USA, 4 tests achieved sensitivities ≥98%, including the Rapidec Carba NP (bioMérieux), the manual Blue Carba, the modified Carba NP and the mCIM.^195,199,200^

Rapid proteomic approaches using MALDI-TOF mass spectrometry are increasingly available and can detect carbapenem hydrolysis directly from colonies or positive blood cultures within a short turnaround time.^201^ Commercial kits such as the MBT STAR^®^-Carba IVD Kit (Bruker Daltonics) or other in-house MALDI-based methodologies have been evaluated and were shown to be reliable in detection of CPE from cultured colonies, with sensitivities ranging from 95%–100% and specificities of 98.2%–100%, and a rapid turnaround time of <1.5 h.^202^ Performance in non-Enterobacterales remains more complex, although tailored approaches have shown promising results for carbapenemase detection in *P. aeruginosa.*^203^

Lateral flow immunoassays have emerged as a practical option for rapid carbapenemase detection. These assays detect carbapenemase enzymes directly through an antibody-antigen reaction and return results rapidly, usually within 5–15 min.^185^ Several platforms demonstrate high sensitivity and specificity for common carbapenemases and can identify multiple enzymes in a single isolate.^204^ The FDA-cleared NG-Test Carba 5 LFA reached 100% sensitivity and 95.3%–100% specificity for the ‘big 5’ carbapenemases in a panel of 185 isolates, returning results within 15 min, and avoiding cross-reaction with other beta-lactamases such as ESBL, AmpC or narrow spectrum OXA enzymes.^205^ Their main limitation lies in restricted target coverage, which may miss less common carbapenemases that are regionally important.

### Genotypic detection methods

Genotypic methods provide rapid and specific identification of known carbapenemase genes and are now widely available in clinical laboratories. Real-time PCR assays, including multiplex platforms, typically target the most prevalent carbapenemases such as KPC, NDM, VIM and OXA-48-like, with inclusion of IMP depending on local epidemiology. Automated sample-to-answer systems (e.g. BD MAX™) can deliver results within hours from cultured isolates or screening swabs,^206^ and some assays combine species identification with resistance gene detection.^207^ Other commercial systems include the Xpert Carba-R assay (Cepheid), which is a cartridge-based system for the GeneXpert instrument, a device that is widely available in hospital settings since the COVID-19 pandemic.^208^ However, costs remain substantial and limited gene panels may miss uncommon or emerging carbapenemases, as well as clinically relevant targets such as GES in *P. aeruginosa* or OXA-23 in *A. baumannii*.

Several emerging platforms that are not yet in routine clinical use may expand access to rapid carbapenemase detection. Isothermal amplification approaches, including loop-mediated isothermal amplification (LAMP) and recombinase polymerase amplification (RPA), reduce equipment requirements and enable rapid DNA amplification at constant temperature.^209^ Similarly, clustered regularly interspaced short palindromic repeats (CRISPR) has recently emerged as a powerful gene editing tool. Xu *et al*.^210^ developed a detection system that incorporates both LAMP, CRISPR-Cas12a elements and a lateral flow immunochromatographic strip for rapid and cost-effective CPE gene detection without requiring bacterial culture. RPA-CRISPR-Cas12a platforms have also been developed that can detect up to five carbapenemases simultaneously at detection limits as low as 1.5 cfu/mL within 30 min.^211^

Whole genome sequencing (WGS) is increasingly incorporated into reference laboratory and public health workflows, though it is not yet part of routine clinical diagnostics in most settings.^212^ WGS can identify carbapenemase genes, characterize more complex resistance mechanisms, and define genetic contexts including plasmids, insertion sequences and integrons. Long-read technologies further enhance resolution of mobile genetic elements and support detailed outbreak investigations.^213^ Despite these advantages, turnaround time, cost and infrastructure requirements currently limit routine clinical deployment in many settings.^214^

As a result, carbapenemase detection now sits at the interface between microbiology and clinical decision making. Realizing this potential requires close collaboration between clinical microbiology laboratories and antimicrobial stewardship programmes, particularly as multiplex molecular assays report genotypic findings whose clinical implications may not be immediately apparent to prescribers (e.g. the therapeutic significance of detecting an IMP gene in *P. aeruginosa* versus an NDM gene in *K. pneumoniae*).^215^ Knowledge of whether resistance is driven by KPC, NDM, OXA-type or other enzymes increasingly shapes antimicrobial selection, interpretation of susceptibility results and stewardship decisions. The expanding diagnostic toolkit described above therefore provides the foundation for effective management of carbapenemase-producing infections, which is discussed in the following section.

## Therapeutics: challenges and treatment options

Effective treatment of infections caused by CPOs remains challenging and increasingly depends on accurate definition of the underlying resistance mechanism. While conventional agents retain a limited role, the introduction of targeted BL/BLI combinations has fundamentally reshaped management, particularly for infections caused by serine carbapenemase producers. Important gaps remain for MBL-producers, *A. baumannii*, and in settings with limited access to newer agents.

### Conventional therapies and their limitations

Polymyxins retain *in vitro* activity against many carbapenem-resistant Enterobacterales, but their clinical utility is constrained by nephrotoxicity, neurotoxicity and inconsistent pharmacokinetic and pharmacodynamic target attainment.^216^ Randomized trials have not demonstrated improved outcomes with colistin-based combination therapy for carbapenemase-producing bloodstream infection or pneumonia,^217,218^ although colistin performed more favourably than anticipated in the CRAB subgroup of CREDIBLE-CR.^219^ Polymyxins may also retain a role in urinary tract infections, where high urinary concentrations can compensate for limited serum exposure. When used, they require optimized dosing and close renal monitoring.

Tigecycline and eravacycline achieve low serum concentrations and are unsuitable as monotherapy for bloodstream infection.^220^ They may be considered for complicated intra-abdominal infections caused by susceptible isolates,^221^ and high-dose tigecycline has been used in selected cases of hospital-acquired or ventilator-associated pneumonia, although data in confirmed carbapenemase producers remain limited.^222^ Randomized trials have demonstrated non-inferiority of eravacycline to carbapenems for complicated intra-abdominal infection,^223,224^ but clinical experience in confirmed CRE remains limited.

Aminoglycosides may be useful for uncomplicated lower urinary tract infection caused by isolates with demonstrated *in vitro* susceptibility, but toxicity and poor tissue penetration limit their role in invasive disease.^225^ Fosfomycin remains an option for uncomplicated cystitis when susceptibility is confirmed, while intravenous fosfomycin may be considered as part of combination therapy for severe infections where access to newer agents is limited,^226^ recognizing the scarcity of high-quality carbapenemase-specific data.

### New agents and emerging resistance mechanisms

The introduction of novel BL/BLI combinations has been the most significant advance in treatment of carbapenemase-producing infections. These agents restore BL activity against organisms producing serine carbapenemases (particularly KPC) and selected OXA-48-like enzymes (Table 4). They lack intrinsic activity against MBLs, unless combined with aztreonam, which is stable to MBL hydrolysis but otherwise vulnerable to co-produced serine beta-lactamases. Optimization of pharmacokinetics and pharmacodynamics is recommended for BL/BLIs and cefiderocol, including the use of extended or continuous infusions, although high-quality outcome data are limited.^227^

**Table 4.** Guideline-based treatment recommendations for infections caused by carbapenemase-producing Gram-negative bacteria

Carbapenemase family	Enterobacterales	*P. aeruginosa*	*A. baumannii*
KPC	**IDSA:** CAZ-AVI, MEM-VAB or IMI-REL preferred; cefiderocol alternative; combination therapy generally not suggested once susceptibility to a preferred beta-lactam is confirmed. **ESCMID:** For severe CRE, suggest MEM-VAB or CAZ-AVI if active *in vitro*; avoid combination therapy when treated with CAZ-AVI, MEM-VAB or cefiderocol; no recommendation for IMI-REL monotherapy.	**IDSA:** Treat as DTR-*P. aeruginosa*: C/T, CAZ-AVI or IMI-REL preferred when susceptible; cefiderocol alternative option. **ESCMID:** Severe DTR-CRPA: suggest C/T if active *in vitro*; insufficient evidence to recommend CAZ-AVI, IMI-REL or cefiderocol.	**IDSA:** Use CRAB guidance (not enzyme-specific). **ESCMID:** Use CRAB guidance (not enzyme-specific).
MBL (NDM/VIM/IMP)	**IDSA:** CAZ-AVI plus aztreonam, or cefiderocol monotherapy, are preferred options. If neither is feasible, aztreonam + MEM-VAB or aztreonam + IMI-REL may be considered in select cases (if OXA-type carbapenemases are not present). **ESCMID:** For severe CRE carrying MBL and/or resistant to new antibiotic monotherapies, suggest aztreonam + CAZ-AVI; cefiderocol conditionally recommended for severe CRE carrying MBL and/or resistant to all other antibiotics.	**IDSA:** New BL/BLI agents (C/T, CAZ-AVI, IMI-REL) lack activity; cefiderocol is the preferred option for DTR-P. aeruginosa with MBL production (if susceptible). **ESCMID:** Phenotype-based CRPA guidance only; if polymyxins/aminoglycosides/fosfomycin are used for severe DTR-CRPA, suggest ≥2 *in vitro*-active drugs (insufficient evidence for cefiderocol or new BL/BLI agents).	**IDSA:** Use CRAB guidance; note durlobactam does not inhibit MBLs. If SUL-DUR is not active *in vitro*, use alternative CRAB regimens guided by susceptibility (e.g. high-dose ampicillin–sulbactam-based combination or other active agents). **ESCMID:** Use CRAB guidance; no MBL-specific recommendation; cefiderocol conditionally recommended against for CRAB.
OXA	**IDSA:** *OXA-48-like*: CAZ-AVI preferred; cefiderocol alternative; MEM-VAB and IMI-REL not suggested. **ESCMID:** *No OXA-48-specific recommendation*; treat as CRE and select an agent active *in vitro* (typically CAZ-AVI). Cefiderocol reserved for CRE with MBL and/or resistant to all other antibiotics.	**IDSA:** OXA enzymes not typically addressed; treat per DTR-P. aeruginosa guidance and *in vitro* susceptibility. **ESCMID:** CRPA phenotype-based guidance only.	**IDSA:** *Acinetobacter acquired OXAs*: preferred SUL-DUR + imipenem or meropenem; if unavailable, high-dose ampicillin–sulbactam as part of combination therapy with ≥1 other active agent (e.g. polymyxin B, minocycline, tigecycline, or cefiderocol). Cefiderocol should be reserved for refractory cases or intolerance and used as part of combination therapy. **ESCMID:** CRAB: ampicillin–sulbactam for HAP/VAP; if resistant, polymyxin or high-dose tigecycline if active. Recommend against polymyxin–meropenem and polymyxin–rifampin; cefiderocol conditionally recommended against.

Recommendations summarize IDSA (2024) guidance on antimicrobial-resistant Gram-negative infections and ESCMID (2022) guidance on the treatment of multidrug-resistant Gram-negative bacteria. ESCMID recommendations are primarily phenotype-based (CRE, DTR-CRPA, CRAB) rather than carbapenemase-specific. All recommendations assume *in vitro* susceptibility, optimized dosing, and appropriate source control.

BL/BLI, beta-lactam/beta-lactamase inhibitor; C/T, ceftolozane–tazobactam; CAZ-AVI, ceftazidime–avibactam; CRAB, carbapenem-resistant *Acinetobacter baumannii*; CRE, carbapenem-resistant Enterobacterales; CRPA, carbapenem-resistant *Pseudomonas aeruginosa*; DTR, difficult-to-treat resistance; DTR-CRPA, difficult-to-treat resistance carbapenem-resistant *P. aeruginosa*; ESCMID, European Society of Clinical Microbiology and Infectious Diseases; HAP, hospital-acquired pneumonia; IDSA, Infectious Diseases Society of America; IMI-REL, imipenem–relebactam; IMP, imipenemase-type metallo-beta-lactamase; KPC, *Klebsiella pneumoniae* carbapenemase; MBL, metallo-beta-lactamase; MEM-VAB, meropenem–vaborbactam; MIC, minimum inhibitory concentration; NDM, New Delhi metallo-beta-lactamase; OXA, oxacillinase; SUL-DUR, sulbactam–durlobactam; VAP, ventilator-associated pneumonia; VIM, Verona integron-encoded metallo-beta-lactamase.

Ceftazidime–avibactam inhibits Class A, Class C and selected Class D beta-lactamases, including KPC and many OXA-48-like enzymes. Surveillance data show consistently high activity against KPC-producing Enterobacterales, and observational studies report improved survival and lower toxicity compared with colistin-based regimens.^228,229^ Although pivotal trials were not limited to carbapenem-resistant infections, clinical trial data support its use across major infection syndromes, including complicated intra-abdominal infection, hospital-acquired and ventilator-associated pneumonia, and complicated urinary tract infection.^230–232^ Ceftazidime–avibactam is therefore a first-line agent for severe infections caused by KPC- or OXA-48-like–producing Enterobacterales when susceptibility is confirmed.

Meropenem–vaborbactam combines a carbapenem with a boronate BLI that has potent activity against KPC and other Class A enzymes but lacks activity against OXA-48-like carbapenemases and MBLs. In the CRE–focused TANGO II trial, meropenem–vaborbactam demonstrated higher clinical cure rates and less toxicity than best available therapy.^233^ Comparative observational studies suggest outcomes similar to ceftazidime–avibactam.^234^ Meropenem–vaborbactam is therefore a suitable treatment option for confirmed KPC-producing infections when susceptibility is demonstrated but should not be used for OXA-48- or MBL-producers.

Imipenem–relebactam inhibits Class A and Class C beta-lactamases but has limited activity against OXA-48-like enzymes and none against MBLs. In RESTORE-IMI-1 it was associated with improved clinical outcomes and reduced nephrotoxicity compared with colistin plus imipenem, although the carbapenem-resistant subgroup was small.^235^ It represents an additional option for KPC-producing infections and for difficult-to-treat *P. aeruginosa*, but should be avoided for OXA-48-like or MBL-producers.

Resistance to novel BL/BLI combinations involves distinct but overlapping mechanisms.^236^ For ceftazidime-avibactam, resistance most commonly arises through mutations in the KPC omega-loop (residues 164–179) and the adjacent 238–243 loop, with substitutions such as D179Y and T243M among the most frequently reported in clinical isolates.^237–240^ These mutations alter the enzyme's substrate profile, increasing ceftazidime hydrolysis while paradoxically restoring carbapenem susceptibility, effectively converting KPC into an extended-spectrum beta-lactamase.^240^ Notably, the structural similarity between ceftazidime and cefiderocol means that many KPC omega-loop variants that confer ceftazidime-avibactam resistance also increase cefiderocol MICs, with cross-resistance observed in over 75% of tested mutants and confirmed in clinical isolates.^241,242^ This cross-resistance limits sequential use of these agents and underscores the importance of susceptibility testing before switching therapy.

For meropenem-vaborbactam and imipenem-relebactam, resistance emerges less frequently during therapy and is primarily driven by loss or disruption of the outer membrane porins OmpK35 and OmpK36, sometimes in combination with increased *bla*_KPC_ copy number.^243,244^ The impact of porin loss is agent-specific, as meropenem traverses porins more readily than imipenem, making meropenem-vaborbactam particularly vulnerable to this mechanism.^244^ Cross-resistance between these agents can occur when porin defects and KPC variants are co-selected, highlighting the importance of susceptibility testing for all available BL/BLI combinations when treatment failure occurs.^239^

Aztreonam–avibactam (or the combination of ceftazidime–avibactam plus aztreonam where the fixed-dose formulation is unavailable) is effective against MBL producers that co-produce ESBL, AmpC, KPC or OXA-48-like enzymes. This strategy exploits the intrinsic stability of aztreonam to MBL hydrolysis while avibactam protects against co-produced serine beta-lactamases. *In vitro* data demonstrate consistent potency against MBL-producing CRE, and pharmacokinetic/pharmacodynamic modelling supports the need for high-exposure regimens.^245^ Observational studies show improved outcomes compared with colistin-based regimens, and Phase 3 data have supported regulatory approval of aztreonam–avibactam for complicated infections.^246^ These regimens are widely considered first-line therapy for NDM-, VIM- and IMP-producing Enterobacterales in most treatment guidelines.^19^ Reduced activity of aztreonam-avibactam has been linked to insertions in PBP3 (*ftsI*), particularly among NDM-producing *E. coli*,^247^ as well as marked overproduction of ESBL or AmpC enzymes that increase aztreonam MICs. Inoculum effects have also been described and may contribute to variable clinical response in severe infections.^248,249^

Cefiderocol is a siderophore cephalosporin with broad Gram-negative activity and stability against most beta-lactamases, including MBLs. Randomized trials have demonstrated non-inferiority to standard comparators for nosocomial pneumonia and complicated urinary tract infection.^233,250^ In CREDIBLE-CR, overall efficacy was similar to best available therapy, although higher mortality was observed in patients with *Acinetobacter* infections, a signal not seen in Enterobacterales.^219^ Subsequent data from the GAME CHANGER trial showed non-inferiority to standard therapy for 14-day mortality in hospital-acquired and healthcare-associated Gram-negative bloodstream infection, including a substantial subset with carbapenem-resistant pathogens. Confidence intervals remained wide in the carbapenem-resistant subgroup,^251^ but superiority over standard of care was not demonstrated. Cefiderocol remains an important option for MBL–producing Enterobacterales and for infections refractory to other novel BL/BLI combinations, provided *in vitro* susceptibility is confirmed using validated methods. Cefiderocol resistance has been associated with alterations in siderophore uptake pathways, including loss or modification of receptors such as CirA, as well as changes in porin or efflux systems and PBP3 insertions.^252^ Reported resistance rates vary substantially by region and species, reinforcing the need for local susceptibility data.

## Considerations in therapy of carbapenemase-producing non-fermenters

Treatment of carbapenemase-producing *P. aeruginosa* differs from CPE because resistance is frequently multifactorial, reflecting the combined effects of carbapenemase production, porin loss, AmpC overexpression and efflux pump upregulation.^19^ Carbapenemase carriage may therefore be only one contributor to the phenotype, and matching therapy to a single enzyme class is often insufficient. In clinical practice, ceftolozane–tazobactam, ceftazidime–avibactam and imipenem–relebactam are preferred when susceptibility is confirmed, but all lack activity against MBL-producers, which represent a substantial proportion of carbapenemase-producing *P. aeruginosa* in many settings.^19^ This has increased reliance on cefiderocol, which retains activity against many MBL-producing strains.^219,253^ For VIM-producing *P. aeruginosa*, aztreonam-avibactam (or ceftazidime-avibactam plus aztreonam) may offer an alternative, as *in vitro* data demonstrate incremental reductions in aztreonam MICs when combined with avibactam, particularly in isolates co-producing serine beta-lactamases.^254,255^ This combination is increasingly used in regions where VIM-producing strains are prevalent, including Southern Europe, Eastern Europe and Latin America, although activity is more modest than against MBL-producing Enterobacterales and clinical outcome data remain limited.^256^ Neither EUCAST nor CLSI has established breakpoints for aztreonam-avibactam against *P. aeruginosa*, complicating susceptibility-guided prescribing.^257^ Polymyxins remain a last-line alternative when other options are exhausted.

The divergence between Enterobacterales and non-fermenter treatment approaches is most pronounced for carbapenemase-producing *A. baumannii*, where infections are typically extensively drug resistant and therapeutic options are limited. Historically, management has relied on high-dose sulbactam-based regimens and polymyxins, reflecting sulbactam's intrinsic activity against *A. baumannii*. Sulbactam-durlobactam represents a major advance, exploiting sulbactam's intrinsic antibacterial activity against *A. baumannii* through direct inhibition of PBP1 and PBP3. Contemporary CRAB isolates produce OXA carbapenemases and other beta-lactamases that degrade sulbactam, but durlobactam, a diazabicyclooctane inhibitor of Class A, C and D serine beta-lactamases, protects sulbactam from hydrolysis and restores its bactericidal activity.^258^ In the ATTACK trial,^259^ sulbactam–durlobactam administered with imipenem was non-inferior to colistin for 28-day mortality and was associated with substantially lower nephrotoxicity. This supports sulbactam-durlobactam as the preferred treatment backbone where available, although its activity does not extend to MBL producers. In settings where sulbactam–durlobactam is unavailable, alternative strategies that replicate this effect are being investigated, including sulbactam combined with avibactam, but clinical data remain limited.^260–262^

Cefiderocol provides an additional option for MBL-producing *A. baumannii*, but non-susceptibility among *A. baumannii* remains substantial with 40.9% of isolates non-susceptible in one study, and NDM producers being most problematic (44.7% non-susceptible).^263^ Furthermore, cefiderocol heteroresistance has been reported in a substantial proportion of CRAB isolates, and may not be detected by standard susceptibility testing.^264^ Although the clinical impact of cefiderocol heteroresistance remains debated, emerging data suggest an association with treatment failure.^264^ Clinical outcome data for cefiderocol treatment of CRAB are mixed. In CREDIBLE-CR, all-cause mortality was higher in the *Acinetobacter* subgroup [21/42 (50%) patients for cefiderocol versus 3/17 (18%) in the best available therapy group]. The subsequent GAME CHANGER trial noted more favourable outcomes: cefiderocol 14-day all-cause mortality was 9% (1 of 11 patients) versus 21% (3 of 14) for standard-of-care; 28-day all-cause mortality was 45% (5 of 11) for cefiderocol versus 50% (7 of 14) for standard-of-care.^251^ Reflecting these uncertainties, current IDSA guidance restricts cefiderocol to refractory or intolerant cases, while ESCMID guidelines conditionally recommend against its routine use for CRAB.^17,19^

### Investigational therapies

Several agents with activity against CPOs are currently in the pipeline including additional novel BL/BLI combinations and drugs designed to bypass classical resistance mechanisms. Cefepime-taniborbactam has activity against KPC-, OXA-48- and MBL-producers (except IMP carbapenemases).^265^ Phase 3 data showed superiority of cefepime-taniborbactam over meropenem for treatment of complicated urinary tract infections (UTIs) and have supported US FDA regulatory submission.^266^ Cefepime-enmetazobactam, recently approved for complicated UTI and (in Europe) nosocomial pneumonia, pairs cefepime with a penicillanic acid sulphone inhibitor of Class A beta-lactamases including KPC and OXA-48.^267^ It also retains activity against some ceftazidime-avibactam-resistant KPC omega-loop variants.

Nacubactam is a diazabicyclooctane with a dual mechanism of beta-lactamase inhibition and direct PBP2 binding. When combined with aztreonam, it may provide an alternative to aztreonam-avibactam for MBL-producing infections.^268^ Ceftibuten-ledaborbactam combines a bicyclic boronate inhibitor of Class A, C and D beta-lactamases with an oral cephalosporin, offering a potential oral step-down option for CPE urinary tract infections.^269^ Cefepime-zidebactam is undergoing clinical evaluation and has broad-spectrum *in vitro* activity including OXA-48-producing Enterobacterales and MBL-producers.^268^ Xeruborbactam, a boronate inhibitor of both serine beta-lactamases and MBLs, is now in early clinical development paired with cefiderocol rather than meropenem as originally conceived,^270^ with Phase 1 data expected imminently (NCT06547554). Adjunctive MBL inhibitors are also advancing and include agents that directly inhibit NDMs (e.g. ANT2681). In parallel, polymyxin derivatives with improved safety profiles (e.g. SPR206, QPX9003) aim to retain broad CPO activity while reducing nephrotoxicity.^271^

Non-traditional approaches are also being explored. Efflux pump inhibitors may restore activity of existing antibiotics in *P. aeruginosa*, where permeability and efflux are major determinants of phenotype.^272,273^ These drugs prevent bacteria from expelling antibiotics, thus increasing their intracellular concentration and activity. In *A. baumannii*, renewed interest in pathogen-specific targets has supported development of agents such as LpxC inhibitors that kill bacteria by causing accumulation of toxic lipid precursors.^274^ Engineered lysins and artilysins capable of breaching the Gram-negative outer membrane provide broad cross-pathogen activity. These enzymes cause lysis by targeting peptidoglycan (an essential cell wall component).^275^

Adjunctive biological therapies highlight a shift towards precision approaches and host-directed therapy. Phage therapy has shown proof-of-concept efficacy *in vitro* and in selected clinical cases, with the potential to spare the microbiota and, in some settings, increase antibiotic susceptibility.^276–278^ Current evidence in carbapenemase-producing infections remains largely limited to case reports and small series, although multiple clinical trials are underway (NCT05453578, NCT05498363, NCT04596319).^276,279–281^ Monoclonal antibodies targeting surface structures or virulence determinants have shown particular promise in hypervirulent carbapenemase-producing *K. pneumoniae*. Results in *P. aeruginosa* have been more modest, reflecting redundancy in virulence pathways. A monoclonal antibody developed against the polysaccharide capsule of a pandrug-resistant, hypervirulent *K. pneumoniae* strain (ST147 with capsule type KL64) showed both *in vitro* bactericidal activity and importantly protected mice from lethal bacteraemia by these KPC/NDM-producing strains.^282^

Immunomodulatory strategies, including interleukin (IL)-7 and immune checkpoint blockade, are pathogen-agnostic and aim to reverse sepsis-associated immune paralysis rather than directly target bacteria.^283,284^ CRISPR-based antibacterial therapy, including phage-delivered systems designed to eliminate resistance genes or selectively kill carbapenemase-harbouring bacteria, represent an emerging platform with early human data now beginning to appear.^285^

## Infection prevention and antimicrobial stewardship

The therapeutic advances outlined above have improved outcomes for many patients with CPO infections, but they have not removed the underlying drivers of spread. Carbapenemase genes remain highly transmissible, colonization is often prolonged, and healthcare networks continue to experience repeated introductions through patient movement and international travel. As a result, infection prevention and antimicrobial stewardship remain central to limiting transmission, protecting vulnerable patient populations and preserving the effectiveness of newer agents.

Infection prevention strategies for CPOs are most effective when implemented as multimodal programmes (Table 5). A 2019 systematic review of quasi-experimental studies found that most successful interventions combined several core elements,^287^ including contact precautions (90% of studies), active surveillance of asymptomatic carriers (80%), routine monitoring of interventions with audit and feedback (80%), patient isolation or cohorting (70%), improved hand hygiene (50%) and enhanced environmental cleaning (40%). Nearly all studies incorporating these components reported reductions in CPO incidence.

**Table 5.** Infection prevention and control measures for CPOs

Intervention/domain	Key actions/examples	Primary target(s)	Setting(s)	Notes on effectiveness/evidence	Key references
Core multimodal IPC bundle (recommended baseline for all CPOs)					
Hand hygiene	WHO 5 Moments; alcohol-based hand rub at point of care; audit with feedback.	All CPOs (transient hand contamination).	All inpatient and high-risk settings; long-term care.	Consistently included in effective bundles; improved compliance reduces MDRO transmission.	^166,167,286^
Contact precautions	Gown and gloves on entry; dedicated equipment; pre-emptive precautions for high-risk admissions.	Direct/indirect contact from colonized or infected patients.	Acute care including ICU; outbreak settings.	Common in successful bundles; depends on adherence and correct donning/doffing.	^167,286,287^
Patient placement and cohorting	Single room with dedicated bathroom where feasible; cohort by organism/mechanism; cohort nursing in outbreaks.	Person-to-person and environmental spread (colonization pressure).	All inpatient; prioritize ICU, oncology, transplant.	Repeatedly linked to outbreak control; prioritize highest-risk patients when rooms are limited.	^166,167,286^
Active surveillance	Risk-based admission screening (e.g. rectal swabs for CPE); contact screening; point-prevalence surveys in outbreaks.	Asymptomatic carriers (primarily CPE); targeted for CRAB/CP-PA in outbreaks.	ICU; haematology/oncology; transplant; dialysis; inter-facility transfers.	Strongly recommended for CPE; reductions seen when coupled with isolation/cohorting.	^167,286,287^
Environmental cleaning	Daily and terminal cleaning of high-touch surfaces; chlorine-based disinfectant (∼1000 ppm); audit tools (fluorescent gel, ATP).	Fomite transmission; persistent contamination (particularly CRAB).	All wards; intensified during outbreaks.	Enhanced cleaning reduces transmission, especially for CRAB; quality improves with auditing.	^167,287^
Enhanced measures for high-risk units and when environmental reservoirs are suspected					
Water/sink/plumbing controls	Reduce splash (rear-draining design); routine drain maintenance; point-of-use filters; avoid body fluid disposal in hand-wash sinks.	Wet-environment reservoirs and splash contamination (particularly CP-PA and CPE).	ICU; burns; transplant; dialysis; outbreak units.	Sustained control usually requires both engineering and behavioural changes.	^167,288^
Rapid diagnostics for IPC	Rapid molecular assays for carbapenemase genes; rapid communication to wards and IPC teams.	Reduce time to isolation/cohorting; support contact tracing.	Microbiology laboratory integrated with IPC; outbreaks.	Shortens time to IPC implementation; benefits depend on linkage to real-time workflows.	^167,286^
WGS for outbreak investigation	Real-time sequencing of patient and environmental isolates; integrate with patient movement data.	Cryptic transmission including plasmid-mediated spread and environmental links.	Outbreaks; high-prevalence units; reference laboratories.	Growing evidence for improved outbreak resolution; benefits depend on rapid turnaround.	^115,143,289^
System-level measures that sustain containment					
CHG bathing	2% chlorhexidine-impregnated cloths in selected units; monitor skin tolerance.	Reduce overall MDRO burden and device-associated infections.	ICU (universal) or selected high-risk wards.	Evidence for CPOs is mixed; appears stronger for reducing colonization than preventing infection.	^290^
Device care bundles	CLABSI/CAUTI/VAP bundles; minimize device days; aseptic insertion.	Device-associated infection pathways.	ICU; surgical wards; dialysis.	Lower HAI rates overall, reducing opportunities for CPO infection and transmission.	^167,286^
Antimicrobial stewardship	Carbapenem-sparing empiric pathways; prospective audit/feedback; governance of newer agents.	Antibiotic selective pressure driving emergence and persistence of resistant Gram-negatives. Particularly relevant for CP-PA, where carbapenem and fluoroquinolone exposure are strong risk factors.	Facility-wide; ICU focus; linked to microbiology.	Associated with reduced CRE/MDRO incidence; complements IPC rather than replacing it.	^167,286^
Education and staff engagement	Regular IPC and PPE training; unit champions; patient/family education.	Human factors and adherence across all measures.	All clinical and support services.	Improves sustained compliance; essential for durable behaviour change.	^167,286^
Inter-facility communication	Transfer forms/flags for CPO status; regional alerts and coordinated public health responses.	Prevent silent importation across healthcare networks.	All transfers (acute, long-term care, dialysis).	Core containment element; reduces network spread when consistently applied.	^291^
Monitoring, audit and feedback	Track hand hygiene/PPE adherence, cleaning quality and screening compliance; unit-level dashboards.	Implementation fidelity and early detection of lapses.	Facility-wide with unit-level reporting.	Integral to multimodal strategies; enables rapid identification of gaps.	^167,287^
Context-specific policies and emerging adjuncts					
Long-term care precautions	Enhanced Barrier Precautions for high-contact care; hand hygiene; device care; transfer communication.	Transmission in residential care while preserving mobility/socialization.	Nursing homes; LTACHs.	CDC approach balances resident well-being with MDRO containment.	^63,269^
Carriage duration policies	Continue contact precautions for known carriers; criteria-based clearance after serial negative screens.	Prevent re-exposure and onward transmission from prolonged carriers.	All inpatient settings including readmissions.	Carriage often persists for months; policies vary and may be prolonged by antibiotic exposure.	^292^
Decolonization (not routinely recommended)	SDD or oral non-absorbable antibiotics; FMT within trials/selected programmes.	Reduce intestinal colonization and transmission risk (primarily CPE).	Research settings; selected centres.	Evidence limited and heterogeneous; not recommended outside trials.	^166,293^

IPC measures are most effective when implemented as a multimodal bundle tailored to local epidemiology, organism and setting.

CAUTI, catheter-associated urinary tract infection; CHG, chlorhexidine gluconate; CLABSI, central line-associated bloodstream infection; CP-PA, carbapenemase-producing *P. aeruginosa*; FMT, faecal microbiota transplantation; HAI, healthcare-associated infection; LTACH, long-term acute care hospital; SDD, selective digestive decontamination; VAP, ventilator-associated pneumonia; WGS, whole-genome sequencing.

These findings are reflected in international guidance, which generally recommends bundled approaches rather than single interventions. The 2017 WHO guidelines emphasize multimodal strategies that consist of at least hand hygiene, surveillance (in particular for CRE), contact precautions, patient isolation and environmental cleaning (e.g. with hypochlorite).^167^ Surveillance should reflect local epidemiology and risk assessment, with potential target populations being patients with prior CPO colonization, contacts of CPO colonized/infected patients and patients with recent hospitalization in CPO endemic settings. The relative contribution of individual bundle elements remains difficult to disentangle, in part because most studies implement several measures simultaneously and also because most data come from outbreak settings and/or in high-risk patient populations (e.g. intensive care units).^294^ Nonetheless, modelling studies suggest that targeted admission screening combined with dedicated staffing or cohort nursing for carriers were the most cost-effective approaches for limiting onward CPE transmission in healthcare networks.^295^

Environmental reservoirs have also become an increasingly important focus, particularly for MBL-producers and *P. aeruginosa*, as discussed above. Hospital sinks, drains and plumbing systems can support persistent biofilms and have been implicated in recurrent transmission.^157,296^ In response, some centres have adopted enhanced sink and drain cleaning protocols, modified sink design to reduce splash, relocated sinks away from patient care areas or trialled waterless models in high-risk units.^160,297–299^ These measures are typically considered adjuncts to, rather than replacements for, standard precautions and surveillance, and their feasibility depends on infrastructure and local outbreak history.

There is ongoing interest in interventions to reduce colonization and thereby decrease transmission risk. Chlorhexidine bathing has clear benefits for some Gram-positive pathogens, but evidence for CPOs is mixed and appears stronger for reducing colonization burden than preventing infection.^300–303^ Selective digestive decontamination using oral aminoglycosides or polymyxins has been evaluated, but European guidelines based on systematic review evidence do not recommend routine decolonization for CRE and cite insufficient evidence for CRAB and extensively drug-resistant *P. aeruginosa.*^304^ Faecal microbiota transplantation has attracted increasing interest as a means of CPO decolonization, but most studies remain small and uncontrolled.^305^ Huttner *et al*.^293^ conducted a trial that involved both selective digestive decontamination and faecal microbiota transplantation but did not find a statistically significant difference.

### Antimicrobial stewardship

Antimicrobial stewardship is central to reducing both the incidence of CPOs and the clinical harm they cause. At a population level, stewardship programmes reduce selection pressure by limiting unnecessary exposure to broad-spectrum agents, particularly carbapenems, and by promoting early de-escalation once microbiology results are available. A large systematic review and meta-analysis found that hospital stewardship interventions were associated with substantial reductions in infections and colonization due to multidrug-resistant Gram-negative bacteria.^306^ More targeted, carbapenem-focused programmes have similarly reported parallel reductions in carbapenem consumption and rates of carbapenem-resistant Gram-negative bacteria in routine hospital practice, supporting stewardship as a component of CPO containment outside outbreak settings.^307^

The strength of the association between antibiotic exposure and CPO acquisition varies by organism. Carbapenem and fluoroquinolone use are particularly well-established risk factors for carbapenemase-producing *P. aeruginosa*, whereas for *K. pneumoniae*, healthcare-associated transmission and colonization pressure may be relatively more important drivers of acquisition than individual antibiotic exposure.^308,309^ Consistent with this, European risk assessments and guidance emphasize stewardship as a core measure to decrease selection pressure and preserve the effectiveness of carbapenems and newer agents used to treat CRE.^166,307^

From a clinical perspective, stewardship aims to improve outcomes by shortening time to effective therapy while reducing avoidable toxicity and emergence of resistance. This is particularly relevant for CPO infections, where delayed active therapy worsens prognosis but indiscriminate escalation accelerates resistance and limits future options. Programmes that integrate rapid diagnostics with stewardship workflows support earlier mechanism-directed therapy and earlier discontinuation of unnecessary broad-spectrum coverage, which can reduce length of stay and complications without compromising safety when implemented with appropriate oversight.^310^ In practice, the most effective stewardship approaches for CPOs combine structured empiric pathways for high-risk patients, prompt reassessment once genotypic or phenotypic carbapenemase results return, and active governance of newer agents through pre-authorization or prospective audit and feedback to preserve effectiveness at the health-service level. In many regions, particularly low- and middle-income countries, access to newer agents remains limited.^311^ Antimicrobial stewardship programmes should therefore support structured access pathways, including restricted-use policies, regional procurement strategies and compassionate-use mechanisms.

## Conclusions and future directions

CPOs are now firmly established as a persistent and evolving global health threat. A central challenge highlighted throughout this Review is the ongoing shift in epidemiology, particularly the increasing dominance of MBLs such as NDM, which are associated with broader resistance profiles and substantially fewer effective treatment options. This shift is occurring alongside continued plasmid-mediated dissemination, expansion into community and environmental reservoirs, and the emergence of strains combining carbapenemase production with enhanced virulence. Together, these trends threaten to erode recent therapeutic gains and underscore the need for approaches that extend beyond management of individual infections.

At the same time, there is a growing disconnect between where CPOs are most prevalent and where advances in diagnostics and therapeutics are most accessible. Many low- and middle-income countries bear a disproportionate burden of CPO infections yet face major constraints in laboratory capacity, surveillance infrastructure and access to newer antimicrobial agents. Even in high-income settings, advanced diagnostics and novel antibiotics are often restricted to reference laboratories or tertiary centres. Addressing this inequity is a critical future priority. Scalable diagnostic platforms, pragmatic infection prevention strategies, sustainable antimicrobial access models and integration of One Health surveillance across human, animal and environmental domains will be essential if global control efforts are to succeed.

Despite these challenges, there are clear opportunities. Diagnostic capabilities are improving rapidly, with increasing availability of molecular assays, lateral flow tests, MALDI-TOF-based approaches and genomic surveillance, enabling earlier and more precise identification of resistance mechanisms. Therapeutic options have expanded meaningfully for serine carbapenemase producers and are beginning to improve for *A. baumannii*, while the pipeline of next-generation BL/BLIs, non-traditional antimicrobials and adjunctive biological therapies continues to grow. The opportunity now lies in integration. Aligning diagnostics with mechanism-directed therapy, embedding antimicrobial stewardship within infection prevention frameworks, and generating high-quality clinical trial data to guide optimal use will be critical. While CPOs remain a formidable challenge, the expanding toolkit described in this Review provides a foundation for more precise, effective and equitable management in the years ahead.
